# 

**Published:** 1888-05-12

**Authors:** 


### HOW TO BE HEALTHY.

ALL that one has to do to maintain perfect health is to carry a buckeye and a potato in the pocket, wear a lung pad, a couple of porous plasters and a magnetic belt, go to bed at nine o'clock every night and get up at six in the morning, taking a cold-water bath and walking seven miles every day before breakfast. Abstain from the use of tobacco, tea, coffee, all manner of intoxicants and rich food of every description. By obeying the above simple rules your life may be made one continual round of giddy health.—*Texas Siftings*.

### Special Notice to Newsagents, Advertisers, and the Trade.

**CHANGE OF OFFICE AND PUBLISHER.**

Notice is hereby given that the Office of The Hospital will henceforth be at 38, Old Jewry (Cheapside end), E.C., to which address all communications should be sent. Mr. A. Doig has been appointed Advertising Agent, and Mr. J. H. S. Hanning, Manager. All Cheques and Post Office Orders should be made payable to J. H. S. Hanning, 38, Old Jewry, E.C. crossed Lloyds, Barnetts, and Bosanquets Bank (Limited).

### To Secretaries and Matrons.

The Editor will be glad to have a copy of the Regulations, and also of every Report issued from the Press of Asylums, Hospitals, Convalescent and Nursing Institutions, as well as those of other Charities, so soon as they are published, and an early copy in duplicate of all appeals for funds. Notices of Annual and other Meetings, Festival Dinners, etc., will be welcomed All such communications should be addressed to *The Editor, The Lodge, Porchester Square, London, W.* Any superintendent, secretary, matron, or other chief official who will regularly send such documents and information will be registered to receive free of cost a cover for binding The Hospital in half-yearly volumes on sending name and address to the publisher.

### BEST TIME FOR TAKING A BATH

ON this point there is considerable difference of opinion. The general idea is that the best and most convenient time for taking a bath is just after getting out of bed in the morning; and there can be no doubt that a cold bath then does act as an invigorating tonic to the system. Dr. Richardson, however, and other eminent authorities on hygiene maintain that this theory is incorrect! and that if for any reason it be impossible to carry out complete ablution twice a day, which is beyond question the best plan, then general ablution is best just before going to bed. "There is no practice," says Dr. Richardson, "more objectionable than to go to bed closely wrapped up in the dust and dirt that accumulats on the surface of the body during the day; nor is there anything I know so conducive to sound sleep as a tepid douche just before getting into bed. I have many times known bad sleepers become the best of sleepers from the adoption of this simple rule. If the body be well sponged over before going to bed, the morning ablution, though it is still better to carry it out, need not of necessity be so general; the face, neck, chest, arms, and hands may be merely well sponged and washed at the morning ablution."

### AMUSEMENTS WITH PROFIT FOR ALL AGES.

**Rules.**—All answers must be addressed to the PRIZE EDITOR, THE HOSPITAL, 38, Old Jewry, Cheapside, London, E.C., not later than the *first* post on Thursday next. The full name and address must in all cases be given, but a *nom de plume* may be added for publication. Only one side of the paper must be written on.

### FOR MEN AND WOMEN.

**Rule.**—Competitors can enter for *all* quarterly competitions, but no competitor may take more than *two quarterly prizes* during the year.

**Acrostic Competition.**

Second quarter commenced February 25th, 1888; ends May 19th, 1888.

A PRIZE of ONE GUINEA will be given to the competitor who gains the highest number of marks for best original acrostics during the ensuing quarter. All acrostics sent in for this competition will be credited according to merit, twelve marks being the highest score given for one acrostic.

A PRIZE of HALF-A-GUINEA to be given to the competitor who gains the highest score for solution of acrostics set. *One mark* only will be given to the competitors who solve all the lights of each acrostic.

Exception will be made in the case of *quotation acrostics*, when *two marks* will be given, *one* for the correct solution of all *lights*, and *one* mark for the *source* of the quotations.

This week credit will be given for

**No. 6.—Original Double Acrostic.**

**Results of No. 5 Original Acrostic.**

Erownie (11), Patience (11), A. M. E. (10).

THE WORD COMPETITION.

Third Quarterly Competition commenced May 5th, 1888; ends July 28th, 1888.

Three prizes of one guinea, 15*s.* and 10*s.* 6*d.* will be given each quarter to the three competitors who obtain the highest number of marks by making the largest number of words derived from the word or phrase set for anatomical dissection; that for this, the *second* week of the quarter, being

"RARE BITS."

OBSERVE.—Proper names, abbreviations, foreign words, words of less than four letters, and repetitions are barred; plurals, and past and present participles, of verbs are allowed. Nuttall's *Standard* dictionary only to be used.

N.B.—All words sent in must be arranged *alphabetically*, with correct total affixed.

**Results of Thirteenth Week.**

*Pastime.*—Ellice (76), Gretta (76), Patience (69), Sym (68), Lightowlers (68), Lauriston (68), Dunce (57), Oliver (51), M. R. (40).

**Results of Second Quarterly Word and Literary Competition.**

*First Prize—Patience* (1,578), Miss C. F. Calise, Craven Road, Newbury.

*Second Prize—Ellice* (1,496), E. E. Bruce, Clatford Vicarage, Andover, Hants.

*Third Prize—Gretta*, for letters and words (1,495), Miss Margaretta Parker, Effingham Villa, Park Lane, Stoke Newington, N.

**Letters from Across the Sea.**

*Oliver* sends the following extract from a letter from Tasmania:—

We are having some very hot weather this summer and very little rain. The weather here always seems to follow the English; so if we hear that this winter is very severe in England, we shall expect the same six months hence. You ask whether we have blackberries. We have, and wish that we had not; for if they once get into the land they are very hard to destroy. The other wild fruits are the wild raspberry, which I think is native, the fruit is not worth picking; the native cherry, which does not grow in this part of the island; the myrtle fruit, which looks like a fungus. Some people also eat the kangaroo-apple, a fruit like a large potato-apple; it grows on a bush about three feet high, and the flower is like a potato-flower. Then there is the pig-face fruit, and the native currant, the plant of which looks like hemlock That is all that I can remember that are eaten, but there are a good many different sorts of bright-coloured berries in the bush.

Extract from a letter from New Orleans:—

You remember Brighton was called the "City of Churches;" well, it cannot be compared to New Orleans. Of course, too, there are few espiscopal or sectarian; nine-tenths are Roman. The first one we visited was the French Cathedral, known as St. Louis. It is a beautiful church, open, as are all Roman Catholic churches, night and day. The ceiling is beautifully painted, the high altar is magnificent, but the chief attraction to me was the altar on the left, called "Notre Dame de Lourdes." It was made of rock, unhewn; and it had a waterfall. Immediately under the apparition was a crucifix, below that a lovely tabernacle, on each side of which were two vases of pink roses. From the altar, *i.e.*, measured to the top of the canopy is 40 ft. While I was there, on the Feast of S. Blasius, hundreds of people came in, and kneeling in turn at the altar, a priest having two lighted candles, made a ⋀ of them, putting one on each side of the person's head, and said a short prayer for deliverance from the "mal au gorge." This ceremony lasted, I was told, about two hours. An old French woman begged us to go, and be prayed for like good Catholics; to her sorrow, we refused. The Jesuits' Church appears the most wealthy; everything in it seems to be the most costly that can be bought. The high altar in this church is not against the wall, but in the centre of the chancel, and there is a chapel behind. The first time we were there a girl could be heard making her confession in the west of the church. She was speaking in the most ordinary tone of voice, and there were scores of people in the church praying and visiting I went again to this church to high mass; the singing was exquisite. Another church is called "Notre Dame de bon Secours," a small but very pretty church, in which some children were being prepared for confirmation. The instruction was in French, and anything more enjoyable than the appearance of the priest cannot be imagined, both he and the children laughed so heartily that they had to hold their sides; their fun spread to us, it was impossible to keep a straight face. The churches of St. Mary and St. Alphonse are close together, the latter noted for its beautiful statuary; the twelve Apostles carved in Parian marble are placed in niches round the high altar. Of course there are many places of historical interest. Coming down the river, about 120 miles from here, is a place called Baton Rouge. We saw sugar and rice plantations stretching for miles and miles; there was the planter's house on each plantation, consisting for the most part of piazzas—many of these buildings are almost palaces; then the sugar house, an immense white building with two big chimneys, and grouped around it 60 or 70 negro huts.

### THE CHILDREN'S CORNER.

PRIZES.

FOR MOST CORRECT ANSWERS TO PUZZLES.

This Commenced October 1st, 1887.

One Pound a Month.

A PRIZE OF THREE GUINEAS

to the Competitor who shall have sent in the largest number of correct or best answers during the twelve months.

**Puzzles—Second Week.**

No. 1. CHARADE.

My *first* beneath your feet in earth
A product lies of greatest worth;
My *next* belongs to me alone,
No other person can it own:
Within my *whole* my first is found
Down in the damp and chilly ground.

No. 2 DOUBLE ACROSTIC.

The initials and finals if rightly you scan
Will give the name of a wonderful man,
Who lived in the reign of a wonderful queen,
Whom no wiser or braver than he was e'er seen.

1. Place these letters at the end,
When "after thoughts" you have to send.
2. A west Indian island fertile and grand,
Found by Columbus, uninhabited land.
3. To this sister o'er the sea,
Joined or parted shall we be.
4. With Robin Hood, in robe of green,
This archer bold was ever seen.
5. Wrath and scorn belong to me;
Never may I dwell with thee.
6. An arctic explorer to all of you known,
Who by courage and hardihood won his renown.

No. 3. ARITHMETICAL PUZZLE.—A boy, having a basket of pears, which he wished to share with three schoolfellows, divided the fruit as follows: To the first he gave half the number in the basket and half a pear over; to the second, half of what remained, and half a pear over; to the third he gave half of the remaining pears, and half a one over, reserving one pear only for himself. Give the total number of pears.

4. TRANSPOSITION.—I am a vessel for holding water; change my vowel and I am something in use in the schoolroom; change it again and I am found on the toilet table; change it once more, and I become a witty saying.

**Results of Fourth Week Puzzles.**

No. 13. DOUBLE ACROSTIC:

W alru S
H awk-mot H
A nacond A
L obste R
E l K.

No. 14. ARITHMETICAL PUZZLE.—There were 28 boys, and the gentleman had 12s. in his pocket.

No. 15. CHARADE.—Horse-man-ship.

No. 16. The quotation refers to Coriolanus.

*All right.*—A. C. K., Bunny, Mount Edgecumbe, Gem. *Three right*—Happy Eliza, Judy, Scrooge, Good Old Killaloe. *Two right.*—Plummy Fluff, Nunkey. *One right.*—McCulloch.

**Results of Seventh Monthly Competition.**

*Bunny* and A. C. K., have each succeeded in gaining full marks during the past month, the latter however is disqualified from receiving any of the prize, having already taken one monthly prize since this competition commenced in October last, we therefore award the whole prize to *Bunny* (Master Wyatt, Lausanne House, 13, Park Crescent, Worthing).

**Conditions.**

I. Every paper sent in must be clearly written, and one side only must be used. All competitors must mark at the head of their papers the number of their competition.

II. Each paper must be signed by the author with his or her real name and address. A *nom de plume* may be added, if the writer does not desire to be referred to by us by his real name. In the case of all prizewinners, however, the real name and address will be published.

III. All communications relating to prize competitions should be adpressed to "The Prize Editor, THE HOSPITAL, 38, Old Jewry, London, E.C." Competitors are requested not to include in letters, etc., to the Prize Editor communications on matters of other business. All letters must be fully prepaid.

IV. The Prize Editor of THE HOSPITAL is the sole judge of the competitions, and his decision is in all cases final and without appeal.

V. The Prize Editor reserves the right to publish any paper sent in, whether it receives a prize or not. He does not hold himself responsible for any MSS. sent, nor can he undertake to return rejected competitions.

VI.—All children resident at school are requested to forward their *home* address in addition to the school one, when entering for the "Children's Corner" Competitions.

**Notice to all Correspondents.**—N.B.—All letters referring to this page, which do not arrive at 38, Old Jewry, Cheapside, London, E.C., by the *first post on Thursdays*, and are not addressed PRIZE EDITOR, will in future be disqualified and disregarded.

No one will be permitted to win the Monthly Prizes twice in any one year, the Annual prize being offered especially to prevent this.

